# Unusual Presentation of an Intraosseous Hemangioma of the Maxilla and Displaced Canine

**DOI:** 10.5005/jp-journals-10005-1203

**Published:** 2013-08-26

**Authors:** Harpreet Kalsi, Jolie Scannell

**Affiliations:** Department of Oral and Maxillofacial Surgery, Queen's Hospital United Kingdom, e-mail: happyharps@hotmail.com; Consultant, Department of Oral and Maxillofacial Surgery, Queen's Hospital, United Kingdom

**Keywords:** Intraosseous hemangioma, Maxilla, Ectopic canine

## Abstract

Intraosseous hemangiomas are benign vascular malformations which are extremely rare in the maxilla, but have been reported in the mandible, zygoma and orbital region. A 12 years old female presented to the oral and maxillofacial department with an ectopically positioned upper left canine in her zygomatic bone and buccal alveolar expansion between the upper left lateral incisor and upper left first premolar.

This case shows the unusual presentation of an intraosseous hemangioma associated with an ectopically migrated upper left canine tooth. It is possible that this lesion caused migration of the tooth. The clinician should be aware of the possibility of this lesion for bony expansile lesions and the importance of radiographic examination in patients who present with delayed eruption of canine teeth.

**How to cite this article:** Kalsi H, Scannell J. Unusual Presentation of an Intraosseous Hemangioma of the Maxilla and Displaced Canine. Int J Clin Pediatr Dent 2013;6(2):124-126.

## INTRODUCTION

Intraosseous hemangiomas (IH) are rare benign vascular tumors of endothelial origin and comprise less than 1% of intraosseous tumors.^1^ The female to male ratio is 2:1 and the peak incidence is between the second and fifth decade of life.^1^ They are usually found in the vertebral column and skull bones, and the facial bones are rarely affected,^2^ with the frontal bone most commonly affected and the zygomatic bone and mandible rarely affected.^2^

IH are usually asymptomatic but can occasionally present as a slow growing hard mass, discomfort, spontaneous hemorrhage, pulsatile sensation or mobile teeth.^1,2^ Radiographic findings can range from a unilocular rounded lesion resembling a cyst, a honeycombed or sunburst appearance or a radiopaque appearance.^1^Differential diagnoses include odontogenic tumors, ameloblastoma, cystic lesions and fibrous dysplasia.^1,3^

This article reports an unusual presentation of an IH in the maxilla with an associated ectopically placed canine tooth in the zygomatic bone.

## CASE REPORT

A 12-year-old female was referred to the Oral and Maxillofacial Surgery Department by an orthodontist regarding an ectopic upper left canine and buccal alveolar expansion from the 22 and 24. On examination there was firm, bony expansion between these teeth with associated root displacement. The 22 and 24 were not mobile. The overlying mucosa was normal and the patient was asymptomatic.

**Fig. 1:**
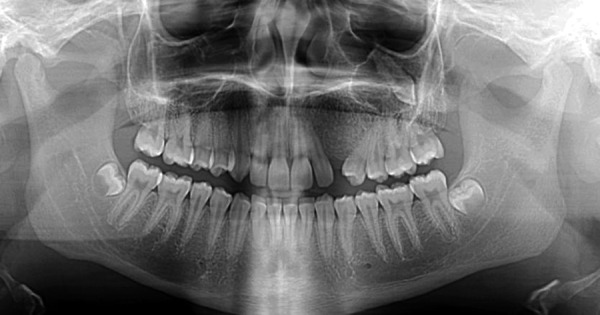
Dental panoramic tomograph showing displacement of 22 and 24 and ectopically positioned 23

**Fig. 2:**
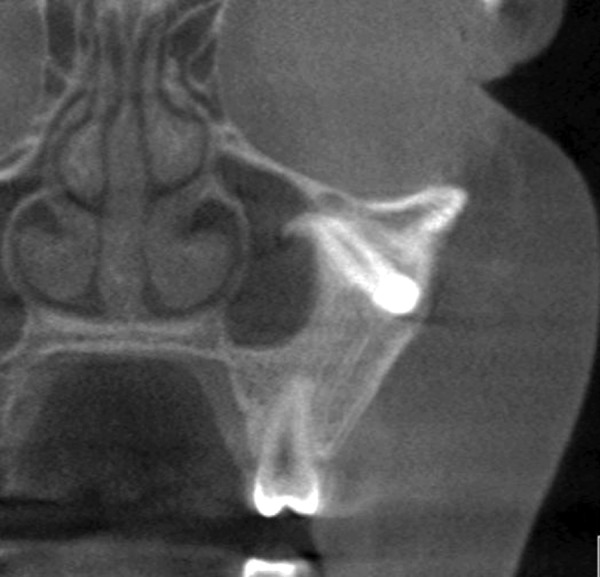
Cone beam CT image showing the position of the ectopic 23 in the zygomatic bone

Radiographic examination revealed an ectopic 23 which appeared to be within the maxillary sinus or a possible large cystic radiolucency surrounding the canine as well as an indistinct area of bony expansion between the 22 and 24 (Fig. 1). There was no evidence of root resorption of the 22 and 24. A cone beam computed tomography (CT) was carried out and showed expanded bone in the 23 region with normal trabecular pattern with intact buccal and palatal cortices, but some buccal cortical expansion. The 23 was located in the left zygomatic bone, with the crown facing buccally and causing dehiscence of the zygomatic bone. The roots were facing toward the left maxillary sinus with the dilacerated apex projecting into the sinus (Fig. 2). The cause of buccal alveolar expansion and migration of the 23 was unclear.

**Fig. 3:**
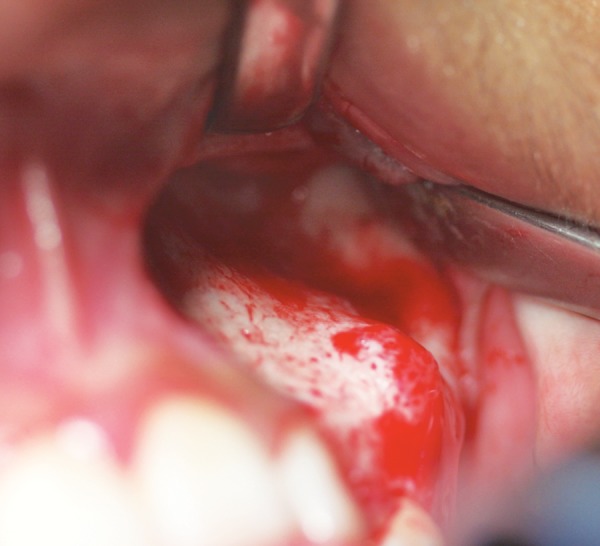
Intraoperative view of bony expansile lesion

The patient had the 23 region explored under a general anesthetic, as well as a bone biopsy of the lesion and a left middle meatal antroscopy. The 23 was not visible during examination of the left maxillary sinus. The expanded buccal bone appeared firm but abnormal looking with slight bleeding (Fig. 3). A differential diagnosis of a fibroosseous lesion was made.

Histopathology results showed vital lamellar bone with uniform osteoblast layer with minimal evidence of resorption. The soft tissue component was a mixture of adipose and highly vascular fibrous tissue. Numerous blood vessels ranged widely in size, some having muscle wall and appeared to be displacing adipose tissue. These finding were compatible with a diagnosis of an IH.

On review, there was no further evidence of bony expansion or active hemorrhage from the lesion. Although the 23 was positioned in the zygomatic bone, there was no cystic evidence and therefore it was not surgically removed. No further investigations were warranted as the patient was asymptomatic. The patient is currently reviewed on a 6-monthly basis with no clinical changes. No further intervention was required for this patient as she was asymptomatic.

## DISCUSSION

IH of the maxilla are extremely rare and to the authors knowledge there has been no published cases of a maxillary IH associated with an ectopically placed tooth, although cases of mandibular IH^1-7^ and maxillary IH have been reported in the literature.^6^ It is possible that this lesion caused migration of the canine tooth.

The prevalence of ectopic canines is reported to be 1.5% and is most commonly positioned in the palate.^8^ The maxillary canines are usually palpable in the buccal sulcus between the ages of 10 to 11, with them erupting usually between the ages of 12 to 13. In this case, the true position of the canine and the presence of an unusual lesion could have been missed without the necessary radiographs.

IH are classified as true benign vasoformative neoplasms or developmental condition of endothelial origin by the World Health Organization.^9^ Clinical features are a firm, painless swelling of the bone, occasional pressure or discomfort, bleeding from the gingival around the lesion and mobile teeth.^3^

Diagnosis can be difficult due to absence of symptoms and unspecific radiological changes, but is usually made by a combination of clinical features, radiographic appearance and histopathology. CT and magnetic resonance imaging (MRI) can aid diagnosis, as well as angiography which can confirm the vascular nature of the lesion by showing its extent and highlighting feeder vessels.^3^

Treatment of IH is not always required and depends upon degree of disfigurement or repetitive bleeding.^1^ Treatment requires interdisciplinary management.^5^ The type of treatment largely depends on the size and location of the lesion and age of the patient and can include surgery, radiotherapy, curettage and embolization.^1^ Embolization of large feeder vessels is usually carried out prior to surgical treatment to reduce intraoperative bleeding. *En bloc* resection is usually performed with ligation of feeder vessels. Reconstruction may involve bone grafts and placement of implants.

## CONCLUSION

Clinicians should be aware of the possibility of an IH in patients presenting with a bony expansile lesion of the maxilla or mandible. Failure to consider this lesion as a differential diagnosis could lead to significant hemorrhage loss during surgical intervention. This case also highlights the importance of radiographic examination in patients who present with delayed eruption of canine teeth.
